# Prevalence and Risk Factors of CKD in Chinese Patients with Periodontal Disease

**DOI:** 10.1371/journal.pone.0070767

**Published:** 2013-08-07

**Authors:** Kejin Liu, Qinghua Liu, Wei Chen, Mengjun Liang, Wei Luo, Xianfeng Wu, Yiping Ruan, Jie Wang, Ricong Xu, Xiaojiang Zhan, Jianwen Yu, Jiaqing Tan, Xiuqing Dong, Jincai Zhang, Xueqing Yu

**Affiliations:** 1 Department of Nephrology, The First Affiliated Hospital, Sun Yat-sen University, Key Laboratory of Nephrology, Ministry of Health, Guangzhou, China; 2 Department of Periodontology, Guangdong Provincial Stomatological Hospital, Southern Medical University, Guangzhou, China; University of Sao Paulo Medical School, Brazil

## Abstract

**Background:**

Periodontal disease is common among adults and is associated with an increasing risk of chronic kidney disease (CKD). We aimed to investigate the prevalence and risk factors of CKD in patients with periodontal disease in China.

**Methods:**

In the current cross-sectional study, patients with periodontal disease were included from Guangdong Provincial Stomatological Hospital between March 2011 and August 2011. CKD was defined as estimated glomerular filtration rate (eGFR) <60 mL/min/1.73 m^2^, the presence of albuminuria, or hematuria. All patients with periodontal disease underwent a periodontal examination, including periodontal probing pocket depth, gingival recession, and clinical attachment level by Florida Probe. They completed a questionnaire and had blood and urine samples taken. The adjusted prevalence of indicators of kidney damage was calculated and risk factors associated with CKD were analyzed.

**Results:**

A total of 1392 patients with periodontal disease were invited to participate this study and 1268 completed the survey and examination. After adjusting for age and sex, the prevalence of reduced eGFR, albuminuria, and hematuria was 2.7% (95% CI 1.7–3.7), 6.7% (95% CI 5.5–8.1) and 10.9% (95% CI 9.2–12.5), respectively. The adjusted prevalence of CKD was 18.2% (95% CI 16.2–20.3). Age, male, diabetes, hypertension, history of CKD, hyperuricemia, and interleukin-6 levels (≥7.54 ng/L) were independent risk factors for reduced eGFR. Female, diabetes, hypertension, history of CKD, hyperuricemia, high level of cholesterol, and high sensitivity C-reactive protein (hsCRP) (≥1.03 mg/L) and TNF-α levels (≥1.12 ng/L) were independently associated with an increased risk of albuminuria. Female, lower education (<high school), and history of CKD were independent risk factors for hematuria.

**Conclusions:**

18.2% of Chinese patients with periodontal disease have proteinuria, hematuria, or reduced eGFR, indicating the presence of kidney damage. Whether prevention or treatment of periodontal disease can reduce the high prevalence of CKD, however, remains to be further investigated.

## Introduction

The prevalence of chronic kidney disease (CKD) is increasing rapidly worldwide, and recognized as a global major public health problem [1]–[3]. Understanding its complex epidemiological features and defining the associated risk factors with CKD would help greatly in prevention of CKD. We have previously reported that high prevalence and low awareness of CKD are present in the adult general population of Guangzhou city in Southern China [4].

Previous studies reported mainly the traditional risk factors for CKD: age, hypertension, diabetes, metabolic syndrome etc. [3], [4]. However, little attentions had been paid to the nontraditional risk factors associated with CKD. Accumulating evidences have demonstrated that inflammation is one of important pathogenic factors in renal injury, and inflammation markers (high-sensitivity C-reactive protein (hsCRP), tumor necrosis factor-α (TNF-α), and interleukin-6 (IL-6)) were positively associated with the prevalent CKD [5]. Our hypothesis is that the population with chronic inflammatory disease may have higher prevalence of CKD.

Periodontal disease is a common chronic inflammatory disease, which occurs in response to Gram-negative bacterial infection from dental plaques [6], with a prevalence ranging from 24.4% to in adults in the United States [7]. Markers of inflammation are elevated in people with periodontal disease [8]. Periodontal disease is associated with an approximately 2.0-fold greater risk of CKD [9], [10]. However, the prevalence of CKD in people with periodontal disease is unknown. We therefore assessed the prevalence of CKD in patients with periodontal disease in Chinese, and evaluated whether the frequency of CKD correlated with the inflammatory markers in this population.

## Methods

### Ethics Statement

All participants gave their written informed consent prior to data collection. Illiterate participants had the information leaflet read out to them and provided a thumb impression. The Human Ethics Committees of The First Affiliated Hospital of Sun Yat-sen University, Guangzhou, China, approved the study.

### Participants

We performed a cross-sectional study (adherence to the STROBE Statement) to investigate the prevalence of CKD and associated risk factors in patients with periodontal disease in Guangzhou City from March 2011 to August 2011 in Southern China. Patients receiving a dental clinical examination, and diagnosed with periodontal disease from Guangdong Provincial Stomatological Hospital were included in this study.

### Screening Protocol and Assessment Criteria

All staff participating in this study received intensive training on proper methods for screening. All participants underwent a thorough periodontal examination by two dentists (Intraclass Correlation Coefficient = 0.817), who were trained following a standard examiner, including periodontal probing pocket depth (PPD), gingival recession, and clinical attachment level (CAL). All measurements were performed and documented in all teeth (third molars were excluded), on six sites per tooth, which were distal buccal (DB), distal lingual (DL), mesial buccal (ML), mesial buccal (MB), straight lingual (L), and straight buccal (B) sites, using the Florida Probe System (Florida Probe®; Florida Probe Corp. Gainesville, FL). Periodontal disease was defined according to the suggested CDC/AAP (Centers for Disease Control -American Academy for Periodontology) case definition for surveillance of periodontitis: ≥ two interproximal sites with ≥3 mm clinical attachment loss and ≥ two interproximal sites with ≥4 mm probing depth (not on the same tooth) or one site with ≥5 mm [11].

All patients with periodontal disease aged between 20 and 75 years were included consecutively. The exclusion criteria for all periodontal disease patients were as follows: edentulousness and a history of disseminated neoplastic disease; liver cirrhosis; acute infectious disease of the oral cavity or salivary glands within 6 months preceding the periodontal examination; antibiotic treatment in the previous 1 month; cytotoxic or immunosuppressive therapy (including steroids) in the previous year, and regular use of non-steroidal anti-inflammatory drugs.

All participants completed a questionnaire documenting their socio-demographic status (e.g. age, sex, education and health insurance), personal and family health history (e.g. diabetes, hypertension, cardiovascular disease [CVD], and hyperlipidemia) and lifestyle behaviors (e.g. smoking and drinking) with the assistance of trained staff. A history of self-reported HBV infection and nephrotoxic medications (e.g. non-steroidal anti-inflammatory drugs and herbs containing aristolochic acid) were also been recorded.

Anthropometric measurements (e.g. weight and height) were obtained using standard protocols and techniques. After removal of heavy clothing and shoes, each subject had body weight and height measurements taken by a calibrated scale. Body mass index (BMI) was calculated as weight (kg) divided by height squared (m^2^). Venous blood was collected after an overnight fast of at least 10 h for measurements of various biomarkers. A clean-catch, midstream, morning urine specimen was collected for dipstick urinalysis (Roche Diagnostics, Mannheim, Germany) and microscopic analysis. Indicators of kidney damage and possible risk factors were examined. All blood and urinary samples were tested in the renal laboratory of The First Affiliated Hospital of Sun Yat-sen University.

### Albuminuria

Urinary albumin and creatinine levels were measured from morning urine using an automatic analyzer (Cobas Integra 400 plus, Roche, Basel, Switzerland). Albumin levels were measured by the immunoturbidimetric method and creatinine levels by Jaffe’s kinetic method. The urinary albumin/creatinine ratio (ACR, mg/g) was calculated on the spot urine albumin and serum creatinine value. Microalbuminuria and macroalbuminuria were defined as an ACR between 30 and 299 mg/g and ≥300 mg/g, respectively [12]. The term “albuminuria” is used to describe the presence of either microalbuminuria or macroalbuminuria.

### estimated Glomerular Filtration Rate (eGFR)

Venous blood was collected after overnight fasting. Serum creatinine (Scr) levels were measured using the same method as urinary creatinine. The eGFR was calculated by using the estimating equation developed by modifying the Modification of Diet in Renal Disease (MDRD) Study equation based on data from Chinese patients with CKD [13]. The resulting calibration equation was (R^2^ = 0.999): calibrated Scr (mg/dL) = 0.893×Scr (mg/dL) +0.39. Reduced renal function was defined as an eGFR<60 mL/min/1.73 m^2^: eGFR = 175×calibrated Scr (mg/dL)^−1.234^×age (year)^−0.179^ (if female×0.79).

### Hematuria

Dipstick testing of morning spot urine samples was conducted (Roche Diagnostics). Periodontal disease patient samples with hematuria of 1+ or greater were reexamined by microscopic analysis within 2 h. Urine samples were centrifuged at 1500 g for 5 min, the supernatant was removed, and the sediment in the remaining supernatant was resuspended. An aliquot (20 µL) of this suspension was placed on a glass slide and examined using subdued bright-field illumination at an original magnification of ×100 and ×400 under a light microscope. Three or more red blood cells per high-power field by microscopy were considered abnormal. Women undergoing menstruation were excluded from the urine test.

### Hypertension and Diabetes Status

Blood pressure was measured three times at 1-min intervals using a mercury sphygmomanometer. The mean of the three readings was calculated unless the difference between readings was >10 mmHg, in which case the mean of the two closest measurements was used. Hypertension was defined as systolic blood pressure ≥140 mmHg, diastolic blood pressure ≥90 mmHg, or self-reported diagnosis of high blood pressure and use of antihypertensive medications in the last 2 weeks irrespective of blood pressure. Fasting blood glucose was measured enzymatically by the glucose oxidase method (Comas Integra 400 plus). Diabetes was defined as fasting plasma glucose ≥7.0 mmol/L (126 mg/dL) and/or 2 h post-prandial plasma glucose ≥11.1 mmol/L (200 mg/dL), the use of insulin, oral hypoglycemic agents, or a previous diagnosis of diabetes.

### Laboratory Measurements

Serum high-sensitivity C-reactive protein (hsCRP) levels were measured using a high sensitivity immunoturbidimetric assay with an autoanalyzer (Cobas Integra 400 plus). Other inflammation biomarkers were measured by high sensitivity multiplex ELISA kits (R&D systems, Minneapolis, MN) in duplicate according to the manufacturer’s protocol in 2011, including IL-6, IL-1β, and TNF-α. The average intra-assay coefficient of variation for IL-6 was 7.3%, TNF-α was 8.5%, and IL-1β was 9.7%. Cytokine levels which below the limit of detection were arranged a value equal to the lower limit of detection for that biomarker. Categories of biomarkers for odds ratio calculations were set at the median for each cytokine: hsCRP, 1.03 mg/L; TNF-α, 1.12 ng/L; IL-6, 7.54 ng/L; and IL-1β, 11.21 ng/L. Serum uric acid, total cholesterol, triglyceride, high-density lipoprotein cholesterol (HDL), and low-density lipoprotein cholesterol (LDL) levels were measured by an autoanalyzer (Cobas Integra 400 plus).

### Statistical Analysis

Data are presented as means ± standard deviation for continuous variables and as proportions for categorical variables. Quantitative variables are summarized as median (IQR). Means were compared by the Student’s t-test or the non-parametric Mann–Whitney test. Qualitative variables were compared by Pearson’s χ^2^ test. We analyzed the association between relevant covariates and indicators of kidney damage with logistic regression models. Covariates analyzed by univariate analysis with *P*<0.05 were included in multivariate analysis.

The overall prevalence of low eGFR, albuminuria, hematuria, and total CKD (defined as the presence of at least one of eGFR values <60 mL/min/1.73 m^2^, albuminuria or hematuria) were reported in total, and by sex and age (<18 years, 18–65 years, and ≥65 years). The prevalence of CKD and indictors of kidney damage was tested for interactions with demographic characteristics (i.e. age group, sex, education and smoking) and with risk factors for CKD (diabetes, hypertension, CVD, hyperlipidemia and hyperuricemia). The prevalence and awareness of diabetes, and the prevalence and control rate of hypertension were reported. Because postprandial glucose results were not available, we reported the awareness of diabetes instead of control of diabetes.

Epidata software version 3.1 (http://www.epidata.dk/download.php) was used for data entry and management. All *P* values were two-sided and *P*<0.05 was considered significant. Analyses were performed with SPSS software, version 19.0 (SPSS, Inc., Chicago, IL).

Data from the 6th China Population Sampling Census in 2011 were used as the standard population (http://www.stats.gov.cn).

## Results

A total of 1392 patients with periodontal disease were invited to participate and 1268 completed the survey and examination. The response rate was 91.1%. Demographic and clinical characteristics of the study population are shown in Table 1. Periodontal disease patients with low eGFR, albuminuria or hematuria were older, and had a higher prevalence of hypertension, history of CVD and CKD than those without indictors of kidney damage. In addition, periodontal disease patients with CKD indicators had a higher percentage of high of CRP (≥1.03 mg/L), high TNF-α (≥1.12 ng/L) and high IL-6 (≥7.54 ng/L) levels than those without indictors of kidney damage. Awareness of diabetes was 89.9% and control of hypertension was 49.4%.

**Table 1:** Characteristics of patients with periodontal disease.

	Total (n = 1268)	Patients without CKD (n = 1009)	Patients with CKD (n = 259)	Indicators of CKD		
				eGFR <60 mL/min/1.73 m^2^ (n = 32)	Albuminuria (n = 98)	Hematuria (n = 157)
Age (years)	53.99±10.85	53.13±10.67	57.32±10.91	66.97±10.44	57.52±10.99	55.13±10.08
Female (%)	845(66.7)	661(65.5)	184(71.0)	7(21.9)	76(77.6)	120(76.4)
≥High school education (%)	914(72.0)	744(73.7)	170 (66.1)	27(84.4)	66(67.3)	94(59.9)
Health insurance coverage (%)	1007(79.4)	800(79.3)	207(79.9)	26(81.3)	84(85.7)	121(77.1)
Diabetes (%)	139(11.0)	100(9.9)	39 (15.1)	11(34.4)	25(25.5)	10(6.4)
Hypertension (%)	241(19.2)	174(17.5)	67 (21.6)	18(56.3)	33(33.7)	37(23.6)
History of CVD (%)	185(14.6)	134(13.3)	51 (19.7)	14(43.8)	21(21.4)	24(15.3)
History of CKD (%)	128(10.1)	86(8.6)	42 (16.3)	9(28.1)	16(16.3)	25(15.9)
Hyperlipidemia (%)	382(30.7)	303(30.4)	79(31.1)	13(40.6)	40(40.8)	40(25.8)
Self-reported HBV infection (%)	96(7.6)	84(8.3)	12(4.7)	1(3.1)	4(4.1)	7(4.5)
Nephrotoxic medications (%)	35(2.8)	30(3.0)	5(2.0)	2(6.3)	1(1.0)	3(1.9)
Current smoker (%)	174(13.7)	145(14.4)	29(11.3)	9(28.1)	10(10.2)	14(8.9)
Habitual drinker (%)	93(7.3)	73(7.2)	20(7.8)	5(15.6)	6(6.1)	10(6.4)
Body mass index (BMI, kg/m^2^)	22.79±3.18	22.78±3.09	22.87±3.53	23.26±3.18	23.83±4.11	22.16±2.90
Hyperuricemia (%)	321(25.3)	239(23.7)	82(31.7)	21(65.6)	37(37.8)	36(22.9)
Cholesterol >5.2 mmol/L	824(65.0)	641(63.5)	183(70.7)	19(59.4)	76(77.6)	108(68.8)
Triglyceride >1.7 mmol/L	562(44.3)	437(43.3)	125(48.3)	15(46.9)	54(55.1)	70(44.6)
HDL≤0.91 mmol/L (%)	98(7.7)	74(7.3)	24(9.3)	6(18.8)	8(8.2)	11(7.1)
LDL≥4.14 mmol/L (%)	182(14.4)	136(13.5)	46(17.7)	5(15.6)	22(22.4)	25(15.9)
hsCRP≥1.03 mg/L (%)^a^	632(49.8)	485(48.1)	147(56.8)	22(68.8)	63(64.3)	76(48.4)
TNF-α≥1.12 ng/L (%)^a^	286(22.6)	213(21.1)	73(28.2)	14(45.2)	30(30.6)	37(23.6)
IL-1β≥11.21 ng/L (%)^a^	608(47.9)	474(47.0)	134(51.7)	22(71.0)	51(52.0)	76(48.4)
IL-6≥7.54 ng/L (%)^a^	608(47.9)	471(46.7)	137(52.9)	24(77.4)	53(54.1)	77(49.0)
Serum creatinine (µmol/L)	61.94±14.76	61.21±12.75	64.77±20.62	105.50±20.73	62.15±20.48	59.64±13.80
ACR (mg/g)	6.87(4.03–13.05)	6.14(3.75–10.68)	14.06(6.44–42.60)	7.20(4.35–19.13)	52.89(39.47–105.01)	8.48(5.30–16.36)
eGFR (mL/min per 1.73 m^2^)	109.92±34.25	110.81±30.66	106.44±45.45	51.67±8.58	108.71±34.49	114.77±48.48

CKD chronic kidney disease; CVD cardiovascular disease; ACR albumin-creatinine ratio; eGFR estimated glomerular filtration rate.

a Value is median (interquartile range).

### Prevalence of Indicators of CKD

#### Estimated GFR

The adjusted prevalence of eGFR<60 mL/min/1.73 m^2^ was 2.7% (95% confidence interval [CI] 1.7–3.7) and the awareness rate was 28.1% in the study population. The prevalence of reduced eGFR increased with age (*P*<0.001). Periodontal disease patients older than 65 years had a higher prevalence of reduced eGFR than those less than 65 years (11.7% vs. 1.4%, *P*<0.001). The prevalence of reduced eGFR was greater in men than that in women (6.2% vs. 0.9%, *P*<0.001). The prevalence of reduced eGFR in periodontal disease patients with diabetes, hypertension, a history of CVD, or hyperuricemia was significantly higher than those without these factors (all *P* values<0.001). The prevalence of reduced eGFR was greatest in periodontal disease patients with diabetes and hypertension (10.8%), whereas in those with neither diabetes nor hypertension, the prevalence was only 2.2% (P<0.001). In addition, periodontal disease patients with a history of CKD had a higher prevalence of reduced eGFR than those without a history of CKD (7.6% vs. 2.2%, *P* = 0.001).

#### Albuminuria

The overall adjusted prevalence of albuminuria was 6.7% (95% CI 5.5–8.1) and the awareness rate was 16.3% in the study population. Microalbuminuria and macroalbuminuria were detected in 6.0% (95% CI 4.7–7.2) and 0.7% (95% CI 0.2–0.9) of periodontal disease patients, respectively. The prevalence of albuminuria increased with age in the study population (*P*<0.001). Periodontal disease patients older than 65 years had a higher prevalence of albuminuria than those less than 65 years (13.7% vs. 5.7%, *P*<0.001). The prevalence of albuminuria was greater in women than that in men (7.8% vs. 4.6%, P = 0.019). The prevalence of albuminuria in periodontal disease patients with diabetes, hypertension, a history of CVD, or hyperuricemia was significantly higher than those without these factors (all *P* values <0.001). Periodontal disease patients with a history of CKD had a higher prevalence of albuminuria than those without a history of CKD (11.0% vs. 6.4%, *P* = 0.040).

#### Hematuria

Hematuria was present in 10.9% (95% CI 9.2–12.5) and the awareness rate was 15.9% in periodontal disease patients. The prevalence of hematuria was greater in women than in men (14.3% vs. 8.8%, *P* = 0.006). Periodontal disease patients with a higher education had a lower prevalence of hematuria than those without a higher education (9.1% vs. 15.8%, *P*<0.001). The prevalence of hematuria in periodontal disease patients with diabetes was significantly higher than that in those without diabetes (11.5% vs. 6.3%, *P* = 0.047).

The prevalence of hematuria was 11.3% in periodontal disease patients with diabetes and hypertension, whereas in those with neither diabetes nor hypertension, the prevalence was only 4.4% (*P*<0.001). Patients with a history of CKD had a higher prevalence of hematuria than those without a history of CKD (17.1% vs. 10.2%, *P* = 0.011).

### Prevalence of CKD

Staging of CKD was performed on the basis of the Kidney Disease Outcome Quality Initiative (K-DOQI) [14]. The adjusted prevalence of indictors of CKD is listed in Table 2. The overall adjusted prevalence of CKD was 18.2% (95% CI 16.2–20.3), of which 11.6% (95% CI 9.9–13.3) were at stage 1, 3.9% (95% CI 2.9–5.0) at stage 2, and 2.6% (95% CI 1.8–3.6) at stage 3. Only one periodontal disease patient with CKD was stage 4 and none was stage 5. The awareness rate of CKD was 16.2% in the study population. There was an increasing trend in the prevalence of CKD with increasing age in all periodontal disease patients (*P*<0.001). The prevalence of CKD in patients with periodontal disease older than 65 years was higher than that in those less than 65 years (32.2% vs. 16.1%, *P*<0.001). Periodontal disease patients with a higher education had a lower prevalence of CKD than those without a higher education (16.6% vs. 22.9%, *P* = 0.005). Patients with diabetes, hypertension, CVD, and hyperuricemia had a higher prevalence of CKD than those without these factors (*P* = 0.020, *P* = 0.002, *P* = 0.048 and *P* = 0.007, respectively). Patients with a history of CKD had a higher prevalence of CKD than those without a history of CKD (29.3% vs. 17.0%, *P*<0.001).

**Table 2:** Adjusted prevalence of indicators of kidney function in the study population.

	Kidney Function		Albuminuria		Hematuria		CKD	
eGFR (mL/min/1.73 m^2^)	Patients (n)	Prevalence (95%CI)	Patients (n)	Prevalence within each eGFR level (95%CI)	Patients (n)	Prevalence within each eGFR level (95%CI)	Patients (n)	Prevalence (95%CI)
≥90	917	63.3(62.0–66.6)	70	6.3(5.0–7.9)	119	11.2(9.3–13.0)	174	11.6(9.9–13.3)
60–89	319	25.7(23.0–28.1)	24	6.9(4.3–9.5)	35	10.3(7.2–13.6)	53	3.9(2.9–5.0)
30–59	31	2.6(1.8–3.6)	3	8.8(0.0–19.4)	3	6.4(0.0–14.3)	31	2.6(1.8–3.6)
15–29	1	0.08(0.00–0.19)	1	–	0	–	1	0.08(0.00–0.19)
<15	–	–	–	–	–	–	–	–
Total	1268	100	98	6.7(5.5–8.1)	157	10.9(9.2–12.5)	259	18.2(16.2–20.3)

CKD chronic kidney disease; eGFR estimated glomerular filtration rate; CI confidence intervals.

### Associated Risk Factors for Indictors of Kidney Damage

Adjusted risk factors associated with indicators of kidney damage are listed in Table 3. After adjusting for a history of CVD, current smoking, hsCRP, HDL, TNF-α, and IL-1β levels, age (*P*<0.001), male (*P*<0.001), diabetes (*P* = 0.048), hypertension (*P* = 0.031), a history of CKD (*P* = 0.047), hyperuricemia (*P* = 0.001), and IL-6 levels (*P* = 0.038) were independent risk factors for eGFR <60 mL/min/1.73 m^2^.

**Table 3:** Adjusted factors associated with indicators of kidney damage.

	Reduced renal function^a^		Albuminuria^b^		Hematuria^c^		CKD^d^	
	OR(95%CI)	*P*	OR(95%CI)	*P*	OR(95%CI)	*P*	OR(95%CI)	*P*
Age(change by 10 years)	3.06(1.90–4.94)	<0.001	–	–	–	–	1.43(1.23–1.66)	<0.001
Female (vs. male)	0.15(0.06–0.39)	<0.001	1.95(1.15–3.31)	0.013	1.65(1.10–2.48)	0.016	1.40(1.01–1.94)	0.043
≥High school education	–	–	–	–	0.50(0.35–0.72)	<0.001	–	–
Diabetes	2.52(1.01–6.30)	0.048	3.18(1.83–5.50)	<0.001	–	–	1.54(1.01–2.38)	0.041
Hypertension	2.55(1.09–5.95)	0.031	1.61(1.01–2.66)	0.045	–	–	–	–
History of CKD	2.63(1.01–7.11)	0.047	2.20(1.20–4.04)	0.011	1.99(1.23–3.24)	0.005	2.29(1.50–3.50)	<0.001
Hyperuricemia	4.34(1.84–10.21)	0.001	1.73(1.07–2.79)	0.025	–	–	1.47(1.06–2.03)	0.021
Cholesterol ≥5.20 mmol/L	–	–	1.76(1.03–2.99)	0.037	–	–	–	–
hsCRP≥1.03 mg/L e	–	–	1.66(1.04–2.66)	0.033	–	–	–	–
TNF–α≥1.12 ng/L^e^	–	–	1.71(1.06–2.78)	0.028	–	–	1.46(1.05–2.03)	0.025
IL–6≥7.54 ng/L^e^	2.70(1.06–6.74)	0.038	–	–	–	–	–	–

a factors included in multivariate logistic regression were: age(change by 10 years), female, diabetes, hypertension, history of CKD, history of CVD, current smoking, hyperuricemia(>422 µmol/L for men, >363 µmol/L for women), hsCRP(≥1.03 mg/L), HDL ≤0.91 mmol/L, TNF-α ≥1.12 ng/L, IL-1β ≥11.21 ng/L and IL-6≥7.54 ng/L.

b factors included in multivariate logistic regression were: age(change by 10 years), female, diabetes, hypertension, hyperlipidemia, history of CKD, BMI (<18.5,18.5–23.9, 24.0–27.9, ≥28.0 kg/m^2^), hyperuricemia (>422 µmol/L for men, >363 µmol/L for women), cholesterol ≥5.2 mmol/L, triglyceride ≥1.7 mmol/L, LDL ≥4.14 mmol/L, hsCRP (≥1.03 mg/L) and TNF-α ≥1.12 ng/L.

c factors included in multivariate logistic regression were: female, education (≥high school vs. <high school), hypertension, history of CKD.

d factors included in multivariate logistic regression were: age(change by 10 years), female, diabetes, hypertension, history of CKD, history of CVD, current smoking, Hyperuricemia (>422 µmol/L for men, >363 µmol/L for women), cholesterol ≥5.2 mmol/L, triglyceride ≥1.7 mmol/L, LDL ≥4.14 mmol/L, hsCRP(≥1.03 mg/L), TNF-α ≥1.12 ng/L, IL-1β ≥11.21 ng/L and IL-6≥7.54 ng/L.

OR odds ratio; CI confidence intervals.

e Value is median (interquartile range).

After adjusting for age, hyperlipidemia, BMI, triglyceride and LDL levels, female (*P* = 0.013), diabetes (*P*<0.001), hypertension (*P* = 0.045), a history of CKD (*P* = 0.011), hyperuricemia (*P* = 0.025), hyper-cholesterol (*P* = 0.037), hsCRP (*P* = 0.033) and TNF-α levels (*P* = 0.028) were independently associated with an increased risk of albuminuria. Female (*P* = 0.016), less than high school education (*P*<0.001) and a history of CKD (*P* = 0.005) were independently associated with hematuria when adjusted for hypertension.

After adjusting for a history of CVD, hypertension, current smoking, cholesterol, triglyceride, LDL, hsCRP, IL-6 and IL-1β levels, age (*P*<0.001), male (*P* = 0.043), diabetes (*P* = 0.041), a history of CKD (*P*<0.001), hyperuricemia (*P* = 0.021), and TNF-α levels (*P* = 0.025) were independent risk factors for CKD.

## Discussion

The present survey showed that the prevalence of CKD was 18.2% in patients with periodontal disease, with low awareness of 16.2%, and inflammation markers (IL-6, hsCRP or TNF-α) are special risk factors for CKD in this population. We know of no other published studies examining the prevalence of CKD in individuals with periodontal disease. All the studies are limited to test the relationship of inflammation markers and CKD in periodontal disease too.

Identification of undiagnosed high-risk individuals is needed to provide the opportunity for early detection and intervention to prevent the onset of CKD. Periodontal disease is a common chronic oral inflammatory disease, and based on the current emerging evidence that periodontal disease is a source of systemic inflammatory burden, it is proposed as a newly nontraditional risk factor for CKD [9]. Furthermore, periodontal disease is prevalent and be ignored in China. According to the third national epidemiological investigation on oral diseases conducted in 2005, gingival bleeding and calculus occur in almost all middle-aged people (77.3% and 97.3%, respectively) and elderly people (68.0% and 88.7%, respectively) [15]. The present study implied that the overall prevalence of CKD in periodontal disease was higher than that detected in the general adult population from the same area of Southern China (12.1%) [4], or from China as a whole (10.8%) [3]. These results are supported by a recent systematic review, which included 5 observational studies, and concluded that there was an increased risk for CKD when periodontitis was present [16]. The findings of this survey suggest the importance of taking into account periodontal disease as a risk factors for potential CKD prevention program in the future, rather than the typical approach of identifying traditional high-risk subgroups for CKD.

When comparing the prevalence of CKD indicators in patients with periodontal disease to other studies, the prevalence of reduced eGFR (2.7%) was comparable to that detected in the general adult population from the same area of Southern China (3.2%) [4], from China as a whole (1.7%) [3], or from American NHANES study (4.7%) [17]. The prevalence of albuminuria (6.7%) in patients with periodontal disease was similar with that (6.6%) among general adults in Guangzhou [4], and lower than 9.2% reported by Zhang et al [3]. We found a 10.9% prevalence of hematuria among periodontal disease patients, which is much higher than that in previous studies [3], [4]. We previous reported a 3.8% prevalence of hematuria among general adults in same area, using same survey protocol. These observations may explain in part by the systemic inflammatory burden caused by the chronic oral Gram-negative bacteria infection in periodontal disease patients. In order to minimize false positive results of hematuria, we tested morning spot hematuria dipsticks first, and performed with a urine microscopic examination by an experienced technician when dipstick results were abnormal. The importance of isolated hematuria in patients with periodontal disease will be revealed in long-term follow-up study.

In addition to traditional risk factors related to CKD, the present study identified risk factors specific to periodontal disease patients. We found that high values of hsCRP and TNF-α level were associated with the albuminuria in patients with periodontal disease; high value of IL-6 level was associated with the reduced eGFR in patients with periodontal disease; high value of TNF-α level were associated with the CKD. In patients with periodontal disease, monocytes and dendritic cells within local periodontal tissues secrete various inflammatory mediators, including IL-1β, IL-6, and TNF-α [18], [19]. Elevation of IL-1 and TNF-α levels induced by periodontitis may play a crucial role in the development of a variety of systemic diseases [20], [21]. Some studies have found that inflammation markers are positively associated with the prevalence of CKD in cross-sectional analyses, and may play an important pathogenic role in CKD by inducing endothelin-1 gene expression and fibrotic gene expression [5], [22].

Periodontal disease is considered as a cause of infection-driven inflammation. The proposed potential mechanisms of our observations are through the infection mediated pathway. Bacterial pathogens causing periodontal disease incite the secretion of inflammatory mediators, including IL-6, TNF-α and CRP. These mediators accelerate atherogenesis, thrombus formation, and platelet aggregation [23]. Atherogenesis of large- and medium-sized renal arteries and arterioles may then lead to ischemia, glomerulosclerosis, and severe renal insufficiency [24]–[26].

Our study has several strengths. All participating staff in present survey received intensive training before the survey, and vigorous quality control programs were conducted to guarantee reliable data collection. Furthermore, in order to improve the diagnosis accuracy of periodontal disease, Florida Probe was used. Which was a periodontal probing system, could precise the electronic measurement to 0.1 mm, and automatically store oral clinical examination information. Electronic recording of the data can eliminate errors that occur when probe tip markings are read visually [27]. This is a significant improvement over conventional manual probes, those are commonly used in other periodontal disease surveys.

There are several limitations in the present study. Firstly, the cross-sectional design of the present study makes it impossible to infer a causal relationship between indicators of CKD and periodontal disease. Secondly, we failed to evaluate the prevalence of indictors of damage and CKD stratified by periodontal disease level. Thirdly, one urine sample examination per patient failed to confirm whether hematuria or albuminuria was persistent. Therefore, the prevalence of CKD may have been overestimated in our study.

In conclusion, this is the first cross-sectional survey of prevalence of CKD in patients with periodontal disease, and important in the light of the unknown prevalence and risk factors of CKD in periodontal disease conditions. The most striking finding in this population was a marked increased prevalence of CKD (18.2%) compared with that in the general population, and the associated high values of hsCRP, TNF-α, and IL-6. Future prospective studies of CKD assessing the role of periodontal disease are required to support the possibility of inclusion of periodontal therapy in preventive targeted approaches of CKD.
